# Cerebrospinal fluid cyclase-associated protein 2 is increased in Alzheimer’s disease and correlates with tau pathology

**DOI:** 10.1186/s40035-024-00462-5

**Published:** 2025-01-16

**Authors:** Alessandro Padovani, Andrea Pilotto, Silvia Pelucchi, Laura D’Andrea, Ramona Stringhi, Federica Gorla, Bahar Aksan, Salvatore Caratozzolo, Alberto Benussi, Alice Galli, Clara Tirloni, Daniela Mauceri, Antonio Canale, Silvana Archetti, Barbara Borroni, Monica Di Luca, Elena Marcello

**Affiliations:** 1 Neurology Unit, Department of Clinical and Experimental Sciences, University of Brescia, Brescia, Italy; 2 Neurology Unit, Department of Continuity of Care and Frailty, ASST Spedali Civili Brescia Hospital, Brescia, Italy; 3 Brain Health Center, University of Brescia, Brescia, Italy; 4 Neurobiorepository and Laboratory of Advanced Biological Markers, ASST Spedali Civili Hospital, University of Brescia, Brescia, Italy; 5 Department of Pharmacological and Biomolecular Sciences, Rodolfo Paoletti”, Università Degli Studi Di Milano, Milan, Italy; 6 Department of Neurobiology, Interdisciplinary Centre for Neurosciences (IZN), Heidelberg University, Im Neuenheimer Feld 366, 69120 Heidelberg, Germany; 7 Neurology Clinic, Trieste University Hospital, Trieste, Italy; 8 Department Molecular and Cellular Neuroscience, Institute of Anatomy and Cell Biology, University of Marburg, Robert-Koch-Str. 8, 35032 Marburg, Germany; 9 Department of Statistical Sciences, University of Padova, Padua, Italy; 10 III Laboratory of Analyses, Brescia Hospital, Brescia, Italy

Synaptic dysfunction represents an early pathological event that precedes neurodegeneration in Alzheimer’s disease (AD), even though the molecular mechanisms that underlie synaptic dysfunction remain to be completely understood [1, 2]. Nonetheless, in vivo synaptic biomarkers are highly relevant as they have the potential to reveal early-stage changes and to track target engagement of specific disease-modifying strategies. A range of cerebrospinal fluid (CSF) synapse-related biomarkers including neurogranin, GAP43, SNAP25, neuregulin-1, PSD-95 and neuronal pentraxin have indeed been reported in AD at variance with other dementing illnesses [1–3].

This work focused on the cyclase-associated protein 2 (CAP2), a postsynaptic actin-binding protein that governs actin cytoskeleton dynamics in dendritic spines. CAP2 is expressed in the brain and in a limited range of tissues. CAP2 downregulation differently affects neuronal and dendritic spine morphology in cerebral cortex and hippocampus [4, 5]. In excitatory neurons, CAP2 regulates cofilin activity and actin cytoskeleton dynamics in spines. CAP2 dimerization is crucial for the long-term potentiation-induced cofilin translocation into spines and structural spine remodelling. This mechanism has been suggested to be altered in AD, with decreased CAP2 expression solely in the hippocampus [5]. Recent studies have shown that cofilin is necessary for Aβ-dependent synaptic dysfunction in primary neurons, and that Aβ-induced activation of cofilin represents an upstream signal impacting on tau/microtubule regulation [6].

Given the biological relationship between CAP2 and AD pathogenesis, we conducted a translational study to evaluate CAP2 level in the CSF of a large sample of AD patients. The clinical cohort included 174 subjects, comprising 110 patients with AD (30 prodromal and 80 mild-to-moderate cases), 20 with dementia with Lewy bodies (DLB), 20 with frontotemporal dementia (FTD) and 24 neurologically healthy controls (HCs) (full methodology in Supplementary Material).

The mean CSF CAP2 level was significantly higher in AD patients (both prodromal and mild to moderate dementia) compared to HCs, DLB and FTD patients (*P* = 0.001) (Fig. 1a, Table S1). In AD and HCs, the CAP2 level significantly correlated with CSF t-tau (*r* = 0.43, *P* < 0.001), p-tau181 (*r* = 0.57, *P* < 0.001) levels, and Aβ42 (*r* = −0.28, *P* = 0.034) in sex- and age-adjusted partial correlation. CAP2 level showed no correlation with MMSE score, either in the overall cohort (*r* = 0.03, *P* = 0.71) or in any of the disease group (AD group, *r* = − 0.14, *P* = 0.18; FTD, *r* = − 0.18, *P* = 0.7; DLB, *r* = − 0.19, *P* = 0.14) (Fig. S1a). In the analyses limited to AD patients and adjusted for core CSF markers, CAP2 level was significantly higher in prodromal AD than in subjects with overt dementia (Table S1). ROC analyses were implemented to assess the discrimination accuracy of CAP2 levels in diagnosing AD compared to HC, FTD and DLB. The AUC-ROC for CSF CAP2 in diagnosing AD was 0.72 (95% CI 0.64–0.80), whereas the CAP2/Aβ42 ratio showed a discrimination accuracy of 0.93 (95% CI 0.88–0.97). Comparison of AUC-ROC between p-tau/Aβ42 (standard for the AD diagnosis) and CAP2/Aβ42 ratios showed no significant difference, but a high correlation between the ratios (*r* = 0.59, *P* = 0.001) (Fig. S1b).

**Fig. 1:**
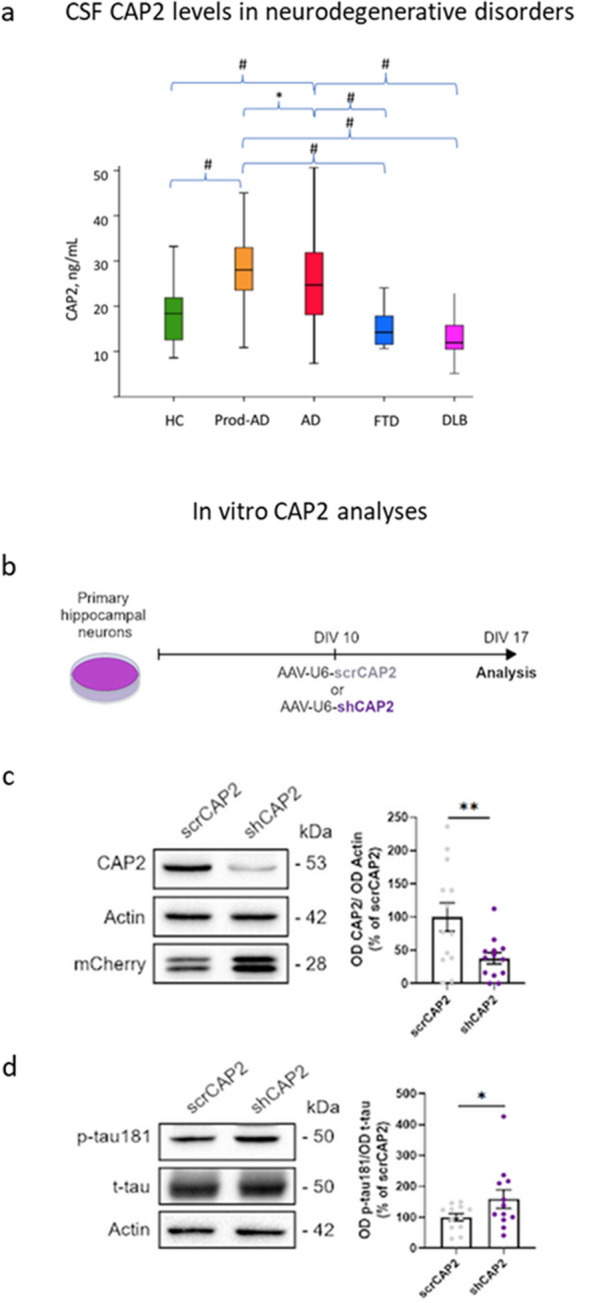
Alterations of CSF CAP2 level in dementia patients and effects of CAP2 downregulation on tau. **a** CAP2 levels in clinical groups. #*P* < 0.001 *P < 0.05. **b-d** CAP2 downregulation promotes tau phosphorylation at Thr181. **b** Experimental paradigm: primary hippocampal neurons at 10 days in vitro (DIV 10) were transduced with rAAV expressing a shRNA targeting *CAP2* (shCAP2) or with a control sequence (scrCAP2). Analysis was performed at DIV 17. **c** Representative Western blots showing the protein levels of CAP2, mCherry to confirm the transduction, and actin as a loading control in homogenate of hippocampal cultures transduced with shCAP2 (or scrCAP2). Quantification of optical density (OD) shows that CAP2 is significantly downregulated (scrCAP2 100 ± 21.34, shCAP2 37.73 ± 8.509; two-tailed paired *t*-test: **P = 0.0040, *n* = 13). **d** Representative Western blots for p-tau181, total tau, and mCherry in lysates of hippocampal neurons transduced with shCAP2 or scrCAP2. The p-tau 181/t-tau ratio was significantly increased when CAP2 was down-regulated (158.8 ± 30.04 versus 100 ± 11.94, Wilcoxon test: *P = 0.0210, *n* = 12). All data are presented as percentage of control (scrCAP2) and expressed as mean ± SE. AD, Alzheimer’s disease; DLB, dementia with Lewy bodies; FTD, frontotemporal dementia; HC, healthy controls; prod-AD, prodromal AD

These findings align with several recent studies showing a steady rise in biomarkers related to synapses in AD, even prior to the onset of axonal degeneration [2, 3]. In agreement with other studies on different synapse-related markers, we confirmed that, compared to DLB and FTD, synaptic parameters are specifically altered in AD, likely associated with altered amyloid-precursor protein metabolism since the early stages. Our results also show a significant difference of CAP2 level between prodromal and mild-to-moderate AD. The higher CAP2 level in prodromal compared to moderate AD cases could possibly be the result of aberrant spine sprouting, which is known to be a potential compensatory mechanism highly active during early phases of the disease. In line with this, in early AD phases, a few synaptic markers, such as NPTX2 are increased, whereas other markers are higher in later phases [2]. Changes in CAP2 level may stem from diverse mechanisms. Increased levels during early disease phases might indicate changes in gene expression or synaptic remodelling by relatively unaffected neurons in response to damage [8].

In subjects with AD, CAP2 level correlated with CSF p-tau181 (*r* = 0.32, *P* = 0.001) and t-tau (*r* = 0.36, *P* < 0.001) but not with CSF Aβ42, with no effect of *APOE* genotype on CAP2 levels (Table S2). Linear regression analyses confirmed the positive correlation of p-tau but not of t-tau levels with CSF CAP2 level in AD subgroups (*P* = 0.03, Table S3). In a subset of 50 patients, NfL assessed by SIMOA (SR-X, QUanterix) appeared to be correlated with CAP2 level in the whole cohort (*r* = 0.325, *P* = 0.025) yet not in the subset of 38 AD patients with available data (*r* = 0.62, *P* = 0.08). The potential link between CAP2 and tau pathology was thus additionally tested in hippocampal cultures containing both neurons and astrocytes (See Supplementary Material). The experiment evaluated the effect of CAP2 downregulation on total and phosphorylated tau levels in hippocampal neurons. To downregulate CAP2, we packaged small-hairpin RNA (shRNA) into recombinant adeno-associated virus (rAAV) particles and transduced them into primary hippocampal neurons (Fig. 1b). The CAP2 protein level was diminished in neurons expressing CAP2 shRNA (shCAP2) compared to neurons transduced with control rAAV (scrCAP2) (Fig. 1c). CAP2 silencing increased the activation of caspase 3 (Fig. S2a, b) without affecting cell viability, astrocyte activation, or levels of synaptic markers, even after Aβ oligomer exposure (Figs. S2 and S3). The downregulation of CAP2 resulted in increased p-tau181 with no effect on t-tau levels (Fig. 1d). These results suggest that CAP2 downregulation contributes to the emergence of key AD neuronal signatures independently of Aβ, such as tau hyperphosphorylation and caspase-3 activation. This finding provides intriguing insights and potential implications for the amyloid cascade hypothesis.

The convergence between clinical and in vitro findings implies a plausible biological connection between CAP2 and tau abnormalities, likely mediated by cofilin [6] or caspase-3 activation [9]. In fact, down-regulation of CAP2 impairs neuronal architecture and spine shape, together with a decrease in synaptic excitatory transmission. In the hippocampal synapses of AD patients and mouse models, previous studies have demonstrated a dramatic increase in cofilin levels, along with a reduction in CAP2 synaptic availability and, accordingly, a decrease in CAP2 dimer formation at synapses [5]. Furthermore, in the hippocampus of AD patients, cofilin precipitates a different pattern of CAP2 monomeric and dimeric forms, suggesting the presence of an ineffective CAP2/cofilin complex in hippocampal synapses that could contribute to the loss of structural plasticity of spines in AD. As a proof of concept, we demonstrated that CAP2 downregulation stimulated caspase-3 activation [9] and tau phosphorylation at Thr181, both AD core alterations. These findings thus further support a link between synaptic dysfunction and tau phosphorylation – possibly linked to cofilin-mediated interaction and pathology at cellular levels in AD. Several lines of evidence suggest that amyloid-induced synaptic dysfunction, maladaptive plasticity and aberrant sprouting might be among the first steps to potentially trigger tau pathology in AD models [1–3].

Further research is needed, including the assessment of CAP2 CSF levels in subjects positive for amyloid but negative for p-tau markers, to comprehensively understand the dynamic trajectories of synaptic changes across the AD spectrum and challenge this hypotheses in vivo. Another limitation of the study was the cross-sectional design, as it did not permit the assessment of the prognostic value of CAP2 at single-subject level. Larger cohorts across AD stages and an independent validation in subjets with amyloid PET are needed to validate these findings, and longitudinal studies could offer insights into the CSF CAP2 trajectory. In fact, actual individual changes in the time-course of MCI towards severe AD are not discernible from our cross-sectional study, lacking multiple time points of observation. Moreover, further studies that incorporate additional CSF markers assessing synaptic integrity, microglia and copathologies [1–3] would prove beneficial in enhancing our understanding of the relationship between CAP2, synaptic function and axonal loss in vivo.

Despite limitations, the study showed that AD is characterized by CAP2 alterations since the prodromal stage and that these alterations are strongly associated with tau-related changes in vivo and in vitro, further arguing for synaptic dysfunction as a central event in the pathogenesis of AD.

## Supplementary Information

**Additional** **file** **1**. **Supplemental Methods**. **Table S1**. Clinical characteristics, core AD and CAP2 CSF levels. **Table S2**. Clinical characteristics, CSF core biomarkers and CAP2 levels in AD patients stratified for APOE genotype. **Table S3**. Multivariable linear regression model for CAP2 CSF levels separately evaluating the correlation with CSF phosphorylated and total tau levels adjusted for clinical and biological variables. **Figure S1**. Correlation between CAP2 levels and MMSE total score in AD patients, and diagnostic accuracy evaluated by mean of ROC AUC for standard core CSF analyses, CAP2 and CAP2/Aβ42 ratios. **Figure S2**. CAP2 downregulation activates Caspase-3 without affecting the percentage of pyknotic nuclei. **Figure S3**. CAP2 downregulation does not affect synaptic markers levels and astrocyte activation.

## Data Availability

The datasets used and/or analysed during the current study are available from the corresponding authors on reasonable request.

## Abbreviations

AD: Alzheimer’s disease
CAP2: Cyclase-associated protein 2
CSF: Cerebrospinal fluid
DLB: Dementia with lewy bodies
FTD: Frontotemporal dementia
HC: Healthy controls
Aβ: β-Amyloid
AUC: Area under the curve
ROC: Reveiver operating characteristic curve
shRNA: Small hairpin RNA
rAAV: Recombinant adeno-associated virus
p-Tau: Phosphorylated Tau
MCI: Mild cognitive impairment
