# Supplementary material for: Cerebrospinal fluid cyclase-associated protein 2 is increased in Alzheimer’s disease and correlates with tau pathology
Source: Transl Neurodegener. 2025 Jan 16;14:1. doi: 10.1186/s40035-024-00462-5 (PMC11736935; doi:10.1186/s40035-024-00462-5)
Supplement: Supplementary file 1 — Additional file 1. Supplemental Methods. Table S1. Clinical characteristics, core AD and CAP2 CSF levels. Table S2. Clinical characteristics, CSF core biomarkers and CAP2 levels in AD patients stratified for APOE genotype. Table S3. Multivariable linear regression model for CAP2 CSF levels separately evaluating the correlation with CSF phosphorylated and total tau levels adjusted for clinical and biological variables. Figure S1. Correlation between CAP2 levels and MMSE total score in AD patients, and diagnostic accuracy evaluated by mean of ROC AUC for standard core CSF analyses, CAP2 and CAP2/Aβ42 ratios. Figure S2. CAP2 downregulation activates Caspase-3 without affecting the percentage of pyknotic nuclei. Figure S3. CAP2 downregulation does not affect synaptic markers levels and astrocyte activation. [file 40035_2024_462_MOESM1_ESM.pdf]

SUPPLEMENTAL MATERIAL

SUPPLEMENTAL METHODS

Patient selection

Consecutive patients diagnosed with AD [1], dementia with Lewy bodies (DLB) [2] or frontotemporal dementia (FTD) [3] who underwent CSF assessment at the Neurology Unit of Brescia between 2019 and 2022, were included in the study. All participants underwent routine blood analyses, standard magnetic resonance imaging (MRI), APOE genotyping, a standardized full cognitive and functional assessment. The following exclusion criteria were applied: (1) cognitive deficits or dementia not fulfilling clinical criteria for neurodegenerative disorders; (2) prominent cortical or subcortical infarcts or brain/iron accumulation at imaging; (3) other neurologic disorders or medical conditions potentially associated with cognitive deficits; (4) bipolar disorder, schizophrenia, history of drug or alcohol abuse or impulse control disorder; (5) recent traumatic events or acute fever/inflammation potentially influencing CSF and plasma biomarkers. AD patients were classified according to the Clinical dementia rating of scale (CDR) (< 1 prodromal AD; ≥ 1 AD dementia). Patients clinically classified as FTD or DLB but with AD-related patterns were excluded a priori from the analyses. For biomarkers comparison, a group of subjects who underwent CSF analyses for headache and no clinical/biomarkers evidence of neurodegenerative disorders (HC, n= 24) were included as healthy control group.

CSF standard biochemical analyses and APOE genotype

Lumbar puncture was performed according to the standardized protocol of the outpatient clinic, from 09:00 to 11:00 in the morning in fasting conditions, after clinical informed written consent was obtained. CSF was collected in sterile polypropylene tubes and gently mixed to avoid gradient effects. CSF was centrifugated and firstly processed for standard biochemical analyses, whereas two milliliters of CSF were stored in cryotubes at −80°C before biomarkers testing. Only patients with

normal routine measures were included in further analyses. CSF Total and Phosphorylated tau at Thr181 (t-tau and p-tau181) and Aβ42 concentrations were measured by Lumipulse (Fujirebio, Ghent, Belgium). Standard cut-off values for AD used by our laboratory are Aβ42 < 650 pg/mL, p-tau > 60 pg/mL, t-tau > 400 pg/mL and p-tau/Aβ42 ratio > 0.9 [4].

CAP2 and NfL level assessment

The CSF samples were diluted at 1:20, and their CAP2 concentration was determined using a commercially available enzyme-linked immunosorbent assay kit (catalog number IK5163; Immunological Sciences, Rome, Italy). This assay has high sensitivity and specificity for CAP2 detection; no significant cross-reactivity or interference between CAP2 and its analogs was observed. pNFL levels were measured on the Simoa SR-X (Quanterix, Lexington, MA, USA) with a single commercial kit analysing both molecules at Spedali Civili of Brescia in a subset of patients. The intra-assay coefficient of variability was 5,73%, and the inter-assay coefficient of variability was 12%. The mean of duplicate assessment was used for final analyses.

IN VITRO METHODS

Animal care

All procedures involving animals were conducted in accordance with the ethical standards of the Institutional Animal Care and Use Committee of the University of Milan (Italian Ministry of Health permit 5247B.N.YOX 2022). Animals were housed in cages with ad libitum access to food and water and 12 h light-dark cycle with controlled temperature of 22°C.

Recombinant adeno-associated viruses (rAAVs)

The sequences used to down-regulate rat CAP2 (shCAP2; CCAAGTCGTCCGAGATGAATGTCCTGGTC) and the scrambled control SCR (scrCAP2;

GCACTACCAGAGCTAACTCAGATAGTACT) were previously cloned in the pAAV-U6>shrna-CaMKIIprom>mcherry vector and validated. [5]

To produce rAAVs HEK 293 were co-transfected via standard calcium phosphate precipitation. Cells were cultured in high-glucose-containing (4.5 g/liter) Dulbecco's Modified Eagle Medium (DMEM; Life Technologies) supplemented with 10% fetal bovine serum, 100 units/ml penicillin and 100 µg/ml streptomycin (Sigma). Culture medium was exchanged to fresh modified Dulbecco medium (IMDM; Life Technologies) containing 5% fetal bovine serum without antibiotics before carrying out the transfection. Packaging of rAAVs was done with helper plasmids pFΔ6, pRV1 and pH21 with either pAAV-scrCAP2 or pAAV-shCAP2. Medium was replaced with fresh DMEM containing 10% fetal bovine serum and antibiotics after transfection. After harvesting, cells pelleted at low speed centrifugation, resuspended in 100 mM NaCl-10 mM Tris-HCl (pH 8.5) and then lysed via 0.5% sodium deoxycholate and freeze-thaw cycles. Viral particles were purified with heparin affinity columns (HiTrap Heparin HP; GE Healthcare) and concentrated with Amicon Ultra-4 centrifugal filter units (Millipore).

Primary neurons, CAP2 downregulation and Western Blot analysis

Primary neurons were isolated from Sprague-Dawley rat hippocampus at embryonic day 18-19 (E18-19) as previously described [5]. To down-regulate CAP2 expression DIV10 primary neurons were transfected using calcium-phosphate method (for Caspase-3 staining) or transduced with an adenoassociated viral vector expressing a shorth hairpin RNA targeting CAP2 (ShCap2) or with a control sequence (scrCAP2) [5]. Analysis was performed at DIV17. Neuronal cells were homogenized at 4°C in an ice-cold buffer with Protease Inhibitor Cocktail (Roche cOmplete™), Ser/Thr and Tyr phosphatase inhibitors (Sigma-Aldrich), 0.32 M Sucrose, 1 mM Hepes, 1 mM NaF, 0.1 mM PMSF, 1 mM $MgCl_2$ using a glass-glass homogenizer. Protein expression was analyzed using standard methodologies. Briefly, protein samples were separated by electrophoresis on acrylamide/bisacrylamide 8% gel and transferred to a nitrocellulose membrane. Primary antibodies

incubation was performed O/N at 4°C. Incubation with the appropriate horseradish peroxidase–conjugated secondary antibodies (Bio-Rad) was conducted for 1 hour at room temperature. The proteins were detected by chemiluminescence using Clarity Western ECL substrate reagent (Bio-Rad cat number 170-5061) and acquired with a ChemiDoc instrument (Bio-Rad). Quantification was conducted with the Bio-Rad ImageLab software.

The following antibodies were used in this study:

- CAP2: 15865-1-AP, Proteintech
- β-Actin: 66009-1-Ig, Proteintech
- mCherry: OAEA00012, Aviva
- tau A0024, DAKO
- p-tauThr181: 12885S, Cell Signaling
- PSD-95: 192757, Abcam,
- VGluT1: NB 75-066, NeuroMab
- SNAP25: 109105, Abcam
- NeuN: MAB377, Millipore,
- Cleaved Caspase-3: 9661, Cell Signaling,
- GFAP: 3670, Cell Signaling.

Amyloid Oligomers Preparation

A$\beta_{1\text{–}42}$ and A$\beta_{42\text{–}1}$ peptides were purchased from Bachem (Bubendorf, Switzerland) and oligomers were prepared according to [6,7] The lyophilized peptides were dissolved in 1,1,1,3,3,3-hexafluoro-2-propanol (HFIP; Sigma, St. Louis, MO, USA) and aliquoted before removing HFIP. A$\beta_{1\text{–}42}$ oligomers (oA$\beta_{1\text{–}42}$) were obtained by incubating at 4 °C for 24 h in Neurobasal medium without Phenol red. The quality of the oligomer preparation was controlled separating the protein onto a 13% Tris-Tricine gels and performing Coomassie staining. Neuronal cultures were treated with either

$oA\beta_{1-42}$ or $A\beta_{42-1}$ at the concentration of 500 nM and, after 24 h, the biochemical or imaging analysis were performed.

Immunofluorescence

Hippocampal neurons were fixed, using 4% PFA + 4% sucrose in PBS, for 10 min at room temperature. Coverslips were then washed three times with PBS and the cells permeabilized with 0,1% Triton X-100 in PBS for 15 min at room temperature. Coverslips were then incubated with 5% Bovine Serum Albumin (BSA) in PBS at room temperature. After 45-60 min neurons were incubated with primary antibodies in 1% BSA-PBS overnight at 4ºC in a humid chamber. After washes with PBS, the incubation with the appropriate secondary antibodies, in 1% BSA-PBS, was performed for 1h at room temperature. The Alexa Fluor dye secondary antibody used was purchased from Thermo Fisher Scientific. The nuclei were stained with Hoechst 33528 20 min at room temperature. The incubation was followed by washes with PBS and mounting on glass slides using Fluoroshield mounting medium (Sigma-Aldrich).

Confocal microscopy

Caspase-3 images were acquired using a Nikon A1R confocal microscope as z-stack series with a 60x oil objective performing sequential acquisition at a resolution of 1024 × 1024 pixels. Cell death analysis and GFAP imaging were performed as a z-stack series using a 40x silicone objective with 0.5x zoom at a resolution of 2048 x 2048 pixels.

Experimental design, patient classification and statistical analysis

Data are presented as mean $\pm$ standard deviation or mean $\pm$ standard error for continuous variables and number (%) for categorical variables. Clinical and demographic characteristics, cognitive assessments, and CAP2 comparisons within diagnostic groups were analyzed using Kruskall-Wallis

Bonferroni-corrected post hoc analyses. Partial correlations, adjusted for age and sex, was applied to investigate the correlations between CAP2 levels and clinical and laboratory variables. Furthermore, linear regression analyses were performed to evaluate the factors associated with CAP2 levels in the entire sample and in AD subjects specifically.

The ability to predict AD was assessed through an overall accuracy analyses of CSF markers, CAP2 and CAP2/Aβ42 ratio using the area under the curve (AUC) of a receiver operating characteristic curve (ROC). DeLong non-parametric method [8] was used to compare different AUC-ROC performances.

The experimental design and analyses accounted for potential biases through randomization and blinding measures in the experimental setting; Western blotting was obtained using computer-assisted imaging (ChemiDoc system and Image lab 4.0 software; Bio-Rad). The optical density (OD) of the proteins was always normalized on actin OD tonormalize for variations in loading and transfer. For imaging analysis, Z-stack images were processed and analyzed using Fiji software (US National Institutes of Health).

All analyses were 2-tailed, and $p < 0.05$ was considered as statistically significant. Statistical analyses were performed with IBM SPSS Statistics version 26 and R for clinical analyses and Prism 6 (GraphPad, La Jolla, CA, USA) for Western Blot and imaging analysis.

SUPPLEMENTARY RESULTS

Table S1. Clinical characteristics, core AD and CAP2 CSF levels.

	**HC (*n*=24)**	**Prodromal AD (*n*=30)**	**Mild to Moderate AD (*n*=80)**	**DLB (*n*=20)**	**FTD (*n*=20)**	***P***
Age, years	63.3 ± 10.1	67.4 ± 8.6	71.1 ± 6.9	64.2 ± 7.0	72.3 ± 4.9	**0.001**[1,2,4,5]
Sex, female %	65.9%	66.7%	59.8%	50.0%	23.8%	**0.05**[5,7,9,10]
Disease duration, years	-	2.0 + 1.3	3.7 + 3.1	3.4 ± 2.3	2.6 ± 2.1	0.12
Comorbidity index, sum	15.5 + 4.4	17.6 + 3.6	19.1 + 3.2	17.5 + 4.5	20.3 + 3.5	0.45
MMSE, score	29.5 ± 1.3	26.9 + 1.4	20.7 + 4.7	21.4 ± 7.8	22.5 ± 3.3	**0.001**[1,2,3,4,5,6,7]
t-tau, pg/ml	248 ± 89	808 ± 458	818 ± 520	370 ± 181	431 ± 447	0.001[1,2,6,7,8,9]
p-tau, pg/ml	25.2 ± 5.6	102 ± 39	113 ± 71	54 ± 27	50 ± 22	0.002[1,2,6,7,8,9]
p-tau/t-tau ratio, pg/ml	0.10 ± 0.7	0.12 ± 0.8	0.14 ± 0.6	0.14 ± 0.8	0.11 ± 0.5	0.34
Aß1-42, pg/ml	1122 ± 352	550 ± 147	523 ± 172	877 ± 288	746 ± 351	0.001[1,2,6,7,8,9]
p-tau/Aß1-42 ratio	0.20 ± 0.18	1.9 ± 1.1	2.2 ± 1.8	0.63 ± 0.26	0.67 ± 0.45	0.001[1,2,6,7,8,9]
**CAP2**						
CAP2, ng/ml	19.1 ± 6.8	29.6 ± 10.4	25.5 ± 9.6	15.2 ± 3.9	14.6 ± 6.6	**0.001**[1,2,5,6,7,8,9]
CAP2/Aß42 ratio	1.9 ± 0.5	5.4 + 5.7	4.8 + 3.1	2.0 ± 1.2	2.8 ± 3.5	**0.001**[1,2,5,6,7,8,9]

Abbreviation: AD, Alzheimer's disease; Aß1-42, Amyloid Beta 1-42 levels; CAP2, Cyclase-associated protein 2; DLB, dementia with Lewy bodies; FTD, frontotemporal dementia; HC, healthy controls; MMSE, MiniMental State Examination; p-tau, phosphorylated Tau levels; t-Tau, total Tau levels. Significant Bonferroni post-hoc comparison between the following groups: [1]HC vs prod AD [2]HC vs AD [3]HC vs DLB [4]HC vs FTD [5]prod AD vs AD [6]prod AD vs DLB [7]prod AD vs FTD [8]AD vs DLB [9]AD vs FTD [10] DLB vs FTD

Table S2. Clinical characteristics, CSF core biomarkers and CAP2 levels in AD patients stratified for APOE genotype.

	**ε3 ε3**	**ε3 ε4**	**ε4 ε4**	***P***
n	**39**	**40**	**11**	
Age, years	69.2 ± 8.6	70.5 ± 7.4	69.3 ± 5.3	0.86
Sex, female %	54.0%	63.6%	54.5%	0.23
Disease duration, years	2.8 + 1.9	2.4 + 1.7	3.6 + 3.6	0.12
MMSE, score	22.6 ± 4.1	22.2 + 4.6	20.8 + 5.1	0.52
Prodromal AD, % (*n*)	77% (*n*=30)	73% (*n*=29)	63% (*n*=7)	0.78
t-tau, pg/ml	854 ± 543	766 ± 356	995 ± 590	0.17
p-tau, pg/ml	125.8 ± 82.1	105.6 ± 43.2	125.5 ± 60.4	0.47
Aß1-42, pg/ml	532 ± 160	584 ± 146.1	464.6 ± 105.7	0.12
p-tau/Aß42 ratio	2.6 ± 2.2	1.8 ± 0.8	2.6 ± 1.0	0.23
CAP2, ng/ml	26.5 ± 8.5	28.0 ± 9.9	24.5 ± 9.0	0.39

Abbreviation: Aß42, Amyloid Beta 1-42 levels; MMSE, MiniMental State Examination; p-tau, phosphorylated Tau levels; T-Tau, total Tau levels.

Table S3 Multivariable linear regression model for CAP2 CSF levels separately evaluating the correlation with CSF phosphorylated and total tau levels adjusted for clinical and biological variables.

	**Beta**	***T***	**Sign.**
**Model CSF p-tau**			
Constant		0.92	0.35
Age	0.181	1.591	0.116
Disease duration	-0.130	-1.207	0.231
sex	-0.166	-1.148	0.143
APOE	0.118	1.012	0.315
Aß1-42 CSF levels	0.092	0.867	0.388
P-tau CSF levels	0.23	2.192	**0.031**
**Model CSF Total Tau**			
Constant		0.88	0.38
Age	0.189	1.612	0.11
Disease duration	-0.145	-1.33	0.18
sex	-0.144	-1.25	0.21
APOE	0.117	0.993	0.32
Aß1-42 CSF levels	0.07	0.657	0.51
P-tau CSF levels	0.183	1.66	0.10

Beta refers to the standardized coefficients of the linear regression model. **Abbreviations:** Aß42, Amyloid Beta 1-42 levels; p-tau, phosphorylated Tau levels; T-Tau, total Tau levels.

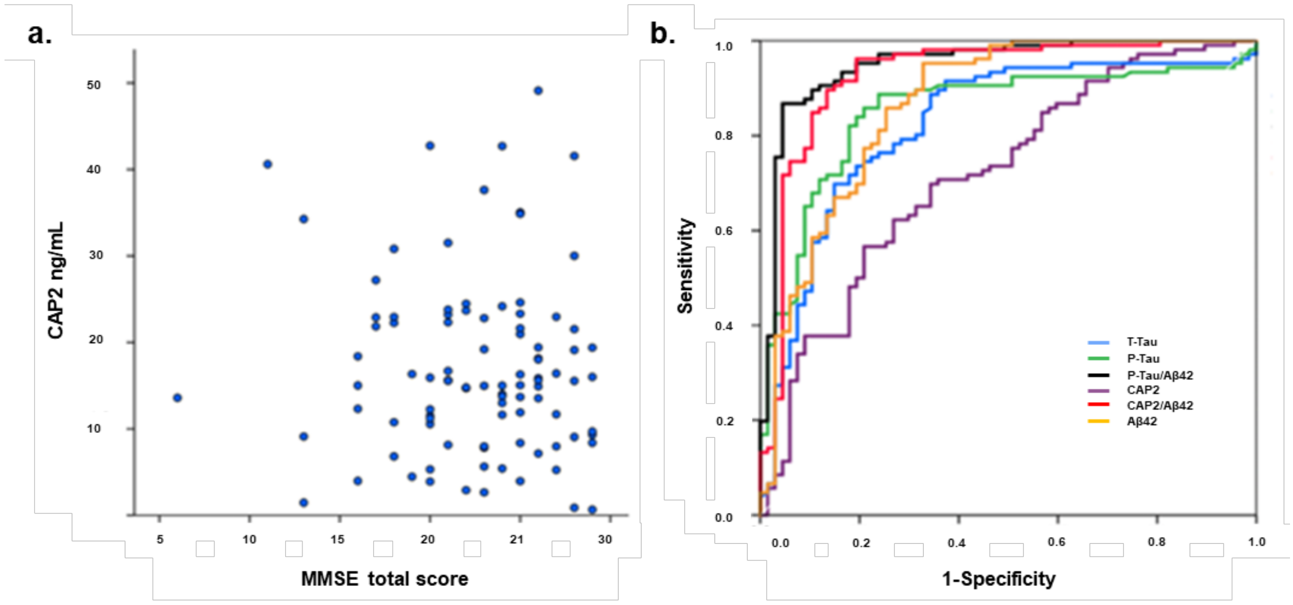

Figure S1. a Correlation between CAP2 levels and MMSE total score in AD patients. **b** Diagnostic accuracy evaluated by mean of ROC AUC for standard core CSF analyses, CAP2 and CAP2/Aβ42 ratios. **Abbreviations:** Amyloid Beta 1-42 levels; p-tau, phosphorylated tau levels; t-tau, total tau levels.

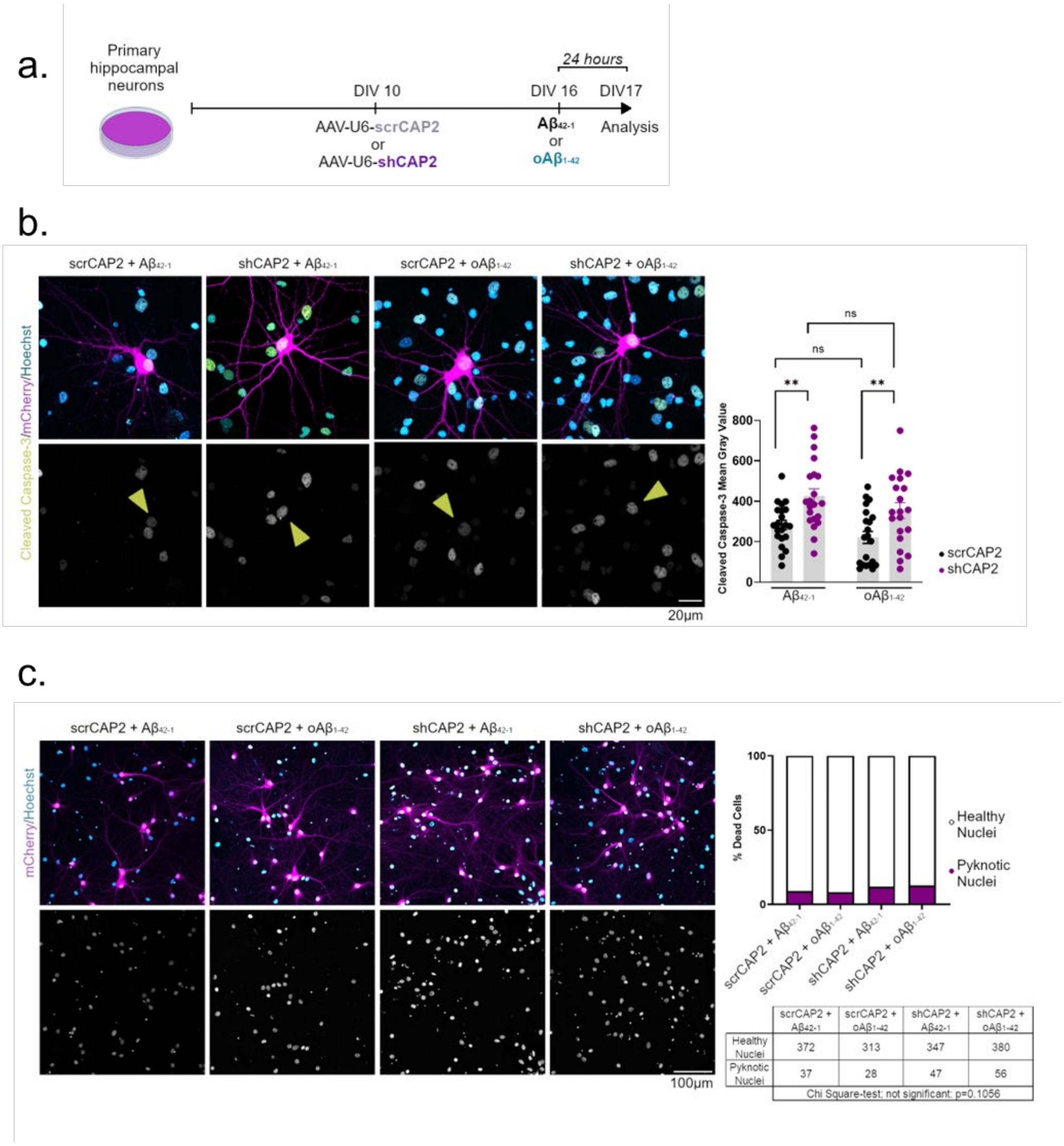

Figure S2. CAP2 downregulation activates Caspase-3 without affecting the percentage of pyknotic nuclei.
a. Experimental paradigm: rat primary hippocampal neurons at 10 days in vitro (DIV 10) were transduced with rAAV **(c)** or transfected with a plasmid **(b)** expressing a shRNA targeting CAP2 (shCAP2) or with a control sequence (scrCAP2). At DIV 16 cells were exposed to oligomers of $A\beta_{1\text{-}42}$ ($oA\beta_{1\text{-}42}$, 500 nM) for 24 hours. As control, we used a peptide with the reverse sequence of Aβ ($A\beta_{42\text{-}1}$). The analysis was performed at DIV17. **b** Representative confocal images of cleaved Caspase-3 (*yellow*) staining of primary hippocampal neurons transfected with shCAP2 (or scrCAP2) plasmids expressing mCherry (*magenta*) as neuronal filler and treated with $oA\beta_{1\text{-}42}$ or the control $A\beta_{42\text{-}1}$ (500 nM, 24 hours); nuclei were detected with Hoechst (*blue*). Scale bar = 20 µm. The downregulation of CAP2 significantly increases nuclear levels of cleaved Caspase-3 (n = 20-21 neurons, two-way ANOVA with Uncorrected Fisher's LSD, shCAP2 - $A\beta_{42\text{-}1}$ vs scrCAP2 - $A\beta_{42\text{-}1}$ **P=0.0025, shCAP2 - $oA\beta_{1\text{-}42}$ vs scrCAP2 - $oA\beta_{1\text{-}42}$**P=0.0054). **c** Representative confocal images of nuclei stained with Hoechst (*blue*) of primary hippocampal neurons transduced with rAAV expressing shCAP2 (or scrCAP2) and mCherry (*magenta*) as neuronal filler and treated with $oA\beta_{1\text{-}42}$ or the control $A\beta_{42\text{-}1}$ (500 nM, 24 hours); Scale bar = 100 µm. The downregulation of CAP2 does not affect the percentage of pyknotic nuclei of CAP2 silenced neurons even upon $oA\beta_{1\text{-}42}$ exposure (Chi-squared test P=0.1056).

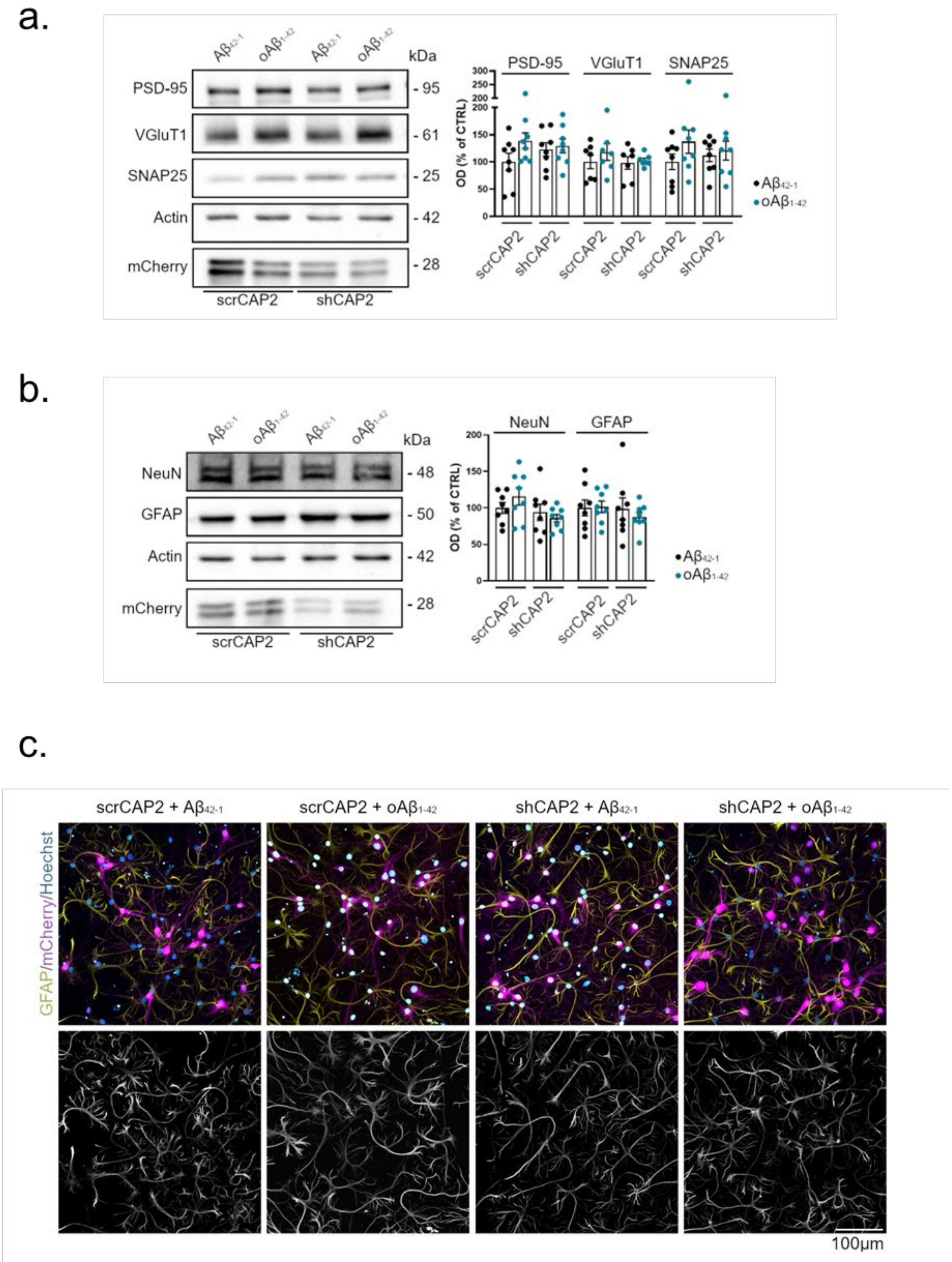

Figure S3. CAP2 downregulation does not affect synaptic markers levels and astrocyte activation. a Representative Western Blot (WB) showing the expression of PSD-95, VGluT1, SNAP25, mCherry (to confirm the transduction) and actin as loading control in homogenate of hippocampal cultures transduced with shCAP2 (or scrCAP2) rAAV and treated with oA$\beta_{1\text{-}42}$ or the control A$\beta_{42\text{-}1}$ (500 nM, 24 hours). Quantification of optical density (OD) shows no changes in PSD-95, VGluT1, SNAP25 (PSD-95, scrCAP2-A$\beta_{42\text{-}1}$=100±15.71, scrCAP2-oA$\beta_{1\text{-}42}$=138.8±14.41, shCAP2-A$\beta_{42\text{-}1}$=122.7±12.4, shCAP2-oA$\beta_{1\text{-}42}$=129.2±12.89; VGluT1, scrCAP2-A$\beta_{42\text{-}1}$=100±12.53, scrCAP2-oA$\beta_{1\text{-}42}$ =117.9±15.33, shCAP2-A$\beta_{42\text{-}1}$=98.09±11.49, shCAP2-oA$\beta_{1\text{-}42}$=102.5±4.07; SNAP25, scrCAP2-A$\beta_{42\text{-}1}$=100±14.25, scrCAP2-oA$\beta_{1\text{-}42}$ =137.1±21.38, shCAP2-A$\beta_{42\text{-}1}$=111.9±11.42, shCAP2-oA$\beta_{1\text{-}42}$=120.7±17.37; two-way ANOVA with Tukey multiple comparisons test, P>0.05, *n*=7-8). **b** Representative WB displaying the levels of NeuN, GFAP, mCherry to confirm the transduction and actin in lysates of hippocampal neurons transduced with shCAP2 (or scrCAP2) rAAV and treated with

$oA\beta_{1-42}$ or the control $A\beta_{42-1}$ (500 nM, 24 hours). Quantification of optical density (OD) shows no changes in NeuN and GFAP when CAP2 is down-regulated and cells treated with $oA\beta_{1-42}$ (NeuN, scrCAP2-$A\beta_{42-1}$=100±7.5, scrCAP2-$oA\beta_{1-42}$ =115.8±11.89, shCAP2-$A\beta_{42-1}$=93.98±11.22, shCAP2-$oA\beta_{1-42}$=85.87±5.32; GFAP, scrCAP2-$A\beta_{42-1}$=100±10.95, scrCAP2-$oA\beta_{1-42}$ =101.5±7.93, shCAP2-$A\beta_{42-1}$=98.71±14.95, shCAP2-$oA\beta_{1-42}$=87.65±6.53; two-way ANOVA with Tukey multiple comparisons test, $P>0.05$, n=8). All data are presented as percentage of control (scrCAP2-$A\beta_{42-1}$) and expressed as mean ± SE. **c** Representative confocal images of astrocytes stained with GFAP (*yellow*) in primary hippocampal cultures transduced with shCAP2 (or scrCAP2) rAAV expressing mCherry (*magenta*) as neuronal filler and treated with $oA\beta_{1-42}$ or the control $A\beta_{42-1}$ (500 nM, 24 hours); nuclei were detected with Hoechst (*blue*). Scale bar = 20 µm. No changes in astrocyte morphology were detected when CAP2 is down-regulated and cells treated with $oA\beta_{1-42}$

References for Supplementary materials:

1. Jack CR, Bennett DA, Blennow K, Carrillo MC, Dunn B, Haeberlein SB, et al. NIA-AA Research Framework: Toward a biological definition of Alzheimer’s disease. Alzheimer’s & Dementia. 2018;14:535–62.

2. McKeith IG, Boeve BF, Dickson DW, Halliday G, Taylor J-P, Weintraub D, et al. Diagnosis and management of dementia with Lewy bodies: Fourth consensus report of the DLB Consortium. Neurology. 2017;89:88–100.

3. Rascovsky K, Hodges JR, Knopman D, Mendez MF, Kramer JH, Neuhaus J, et al. Sensitivity of revised diagnostic criteria for the behavioural variant of frontotemporal dementia. Brain. 2011;134:2456–77.

4. Pilotto A, Parigi M, Bonzi G, Battaglio B, Ferrari E, Mensi L et al. Differences Between Plasma and Cerebrospinal Fluid p-tau181 and p-tau231 in Early Alzheimer's Disease J Alzheimers Dis. 2022;87(3):991-997.

5. Pelucchi S, Vandermeulen L, Pizzamiglio L, Aksan B, Yan J, Konietzny A, et al. Cyclase-associated protein 2 dimerization regulates cofilin in synaptic plasticity and Alzheimer’s disease. Brain Commun. 2020;2:fcaa086.

6. Stine WB, Dahlgren KN, Krafft GA, LaDu MJ (In vitro characterization of conditions for amyloid-beta peptide oligomerization and fibrillogenesis. J Biol Chem 278:11612–11622.

7. Marcello E, Musardo S, Vandermeulen L, Pelucchi S, Gardoni F, Santo N et al. Amyloid-β Oligomers Regulate ADAM10 Synaptic Localization Through Aberrant Plasticity Phenomena. Mol Neurobiol. 2019 Oct;56(10):7136-7143. doi: 10.1007/s12035-019-1583-5.

8. DeLong ER, DeLong DM, Clarke-Pearson DL. Comparing the areas under two or more correlated receiver operating characteristic curves: a nonparametric approach. Biometrics. 1988;837–45.
